# Aerobic Exercise Alleviates Oligodendrocyte Injury and Ferroptosis-Related Features in MPTP-Induced Parkinsonian Mice with Improved Neuropathological Phenotypes

**DOI:** 10.3390/brainsci16090904

**Published:** 2026-08-24

**Authors:** Min Yan, Sen Zhang, Zigui Zhou, Changzhi Yang, Xuewen Tian, Peijie Chen

**Affiliations:** 1 School of Sports and Health, Tianjin Sport University, Tianjin 301617, China; 202520200585@stu.tjus.edu.cn; 2 Academy of Sports Sciences, Shandong Sport University, Jinan 250102, China; 3 School of Sports and Health, Shanghai Sport University, 650 Qingyuan Road, Shanghai 200438, China

**Keywords:** Parkinson’s disease, ferroptosis, oligodendrocytes, aerobic exercise

## Abstract

**Highlights:**

**What are the main findings?**

- Single-nucleus sequencing and spatial transcriptomic analyses revealed reduced oligodendrocyte markers and significant enrichment of ferroptosis-related pathways in the substantia nigra-enriched midbrain region of Parkinson’s disease mice.
- Six weeks of aerobic exercise restored oligodendrocyte-related markers, enhanced NRF2–GPX4 antioxidant signalling, alleviated ferroptosis-related stress, and improved neurological deficits.

**What are the implications of the main findings?**

- Oligodendrocyte injury and ferroptosis-related stress may contribute to Parkinson’s disease pathology and represent potential therapeutic targets.
- Aerobic exercise may protect the oligodendrocyte–neuronal unit by strengthening antioxidant defence, supporting its use as a non-pharmacological intervention for Parkinson’s disease.

**Abstract:**

**Objectives:** The pathological progression of Parkinson’s disease (PD) involves alterations across multiple neural cell types, and glial–neuronal communication substantially influences neuronal function. Oligodendrocytes (OLs) have been implicated in PD pathology, but the underlying regulatory mechanisms remain incompletely understood. **Methods:** In this study, single-nucleus RNA sequencing and spatial transcriptomics were used to characterize OL-associated changes and explore potentially relevant mechanisms in the substantia nigra pars compacta (SNpc) of MPTP-induced parkinsonian mice. Molecular validation was subsequently performed in an exercise intervention cohort. **Results:** These analyses revealed a significant reduction in OL abundance in the SNpc, accompanied by enrichment of ferroptosis-related pathways. Aerobic exercise partially restored the expression of the OL marker gene Plp1 and the antioxidant pathway-related molecules Nrf2 and Gpx4, while reducing ferroptosis-related oxidative stress. These changes were associated with improvements in PD-like pathological phenotypes. Exploratory untargeted metabolomics further identified candidate alterations in metabolites and pathways related to redox homeostasis, energy metabolism, and myelin-associated processes after MPTP treatment and exercise intervention. **Conclusions:** Collectively, exercise-associated improvements in MPTP-induced PD-like phenotypes coincided with reductions in OL/myelin-related injury and ferroptosis-related stress. These findings suggest that OL-associated ferroptosis-related stress may represent one of several processes contributing to neuronal injury in PD and may be responsive to aerobic exercise. This study provides a theoretical basis for further investigation of exercise-based rehabilitation strategies and potential therapeutic targets for PD.

## 1. Introduction

Parkinson’s disease (PD), the second most common neurodegenerative disorder worldwide, remains incurable. It has been estimated that, by 2040, 12 to 17 million people worldwide will be living with PD [1]. The pathological hallmarks of PD include degeneration and loss of dopaminergic (DA) neurons in the substantia nigra pars compacta (SNpc), together with the accumulation of Lewy bodies. Clinical manifestations include both typical motor and non-motor symptoms [2,3], which substantially impair patients’ quality of life.

Oligodendrocytes (OLs) are responsible for myelin formation and the support of efficient neural signal conduction in the central nervous system [4]. Recent studies have shown that OL injury and demyelination are present in patients with PD and may precede the onset of motor symptoms [5,6,7,8], suggesting that OLs may contribute to PD pathogenesis. However, the underlying mechanisms remain poorly defined. OLs contain among the highest levels of iron in the central nervous system and are particularly sensitive to ferroptosis [9,10,11]. Quantitative analyses have shown that iron levels in Olig2-positive OLs are approximately 2.5-fold higher in the brains of patients with PD than in controls [12]. Ferroptosis has been proposed as a potential contributor to PD pathogenesis. However, existing studies have largely focused on ferroptosis in DA neurons, whereas the role of OL ferroptosis in PD remains poorly understood. Furthermore, crosstalk between ferroptosis and neuroinflammation may be involved in PD progression, with shared regulatory molecules such as Nrf2 and Gpx4 [13]. Inflammatory responses may therefore provide supportive indicators of ferroptosis-related stress in OLs, suggesting that targeting iron metabolic imbalance in OLs may represent a potential strategy for PD intervention.

Current treatments for PD are primarily pharmacological or surgical, but both approaches have important limitations [14]. Therefore, additional intervention strategies are needed. In recent years, exercise has increasingly been recommended as an adjunctive intervention for PD. Long-term exercise has been associated with a lower incidence of PD [15], and exercise has been reported to improve PD-related symptoms partly through modulation of ferroptosis [16,17]. However, whether exercise affects ferroptosis-related processes in OLs remains unclear [18]. Exercise has also been shown to promote OL proliferation and myelin repair [19,20]. However, whether these effects involve suppression of ferroptosis-related stress in OLs remains to be determined. Based on these considerations, the present study was designed to investigate whether aerobic exercise is associated with neuroprotective effects in PD mice and whether OL ferroptosis-related stress may be involved.

## 2. Materials and Methods

### 2.1. Laboratory Animals and Grouping

Animals: The experiment was performed using 8-week-old male SPF-grade C57BL/6J mice weighing 20–25 g, which were purchased from Wuhan Yunklon Animal Co., Ltd. (Wuhan, China) All procedures were approved by the relevant ethics committee (approval no. 2024017, April 2024). Before the experiment, mice were acclimatized for 1 week in individual cages under controlled conditions: ambient temperature, 20–26 °C; relative humidity, 40–70%; and a 12 h light/dark cycle. Efforts were made to minimize animal use and suffering. General health status, including body weight, food and water intake, fur condition, locomotor activity, and abnormal behaviour, was monitored daily.

Grouping: The sample size was calculated using G*Power software (version 3.1.9.7), and a total of 45 mice were included in this study. Mice were randomly assigned to the control group (Ctrl group, *n* = 15) or the MPTP model group (*n* = 30). Mice in the Ctrl group were intraperitoneally injected with an equal volume of normal saline and served as sham-injected controls, whereas mice in the MPTP model group were intraperitoneally injected with 1-methyl-4-phenyl-1,2,3,6-tetrahydropyridine (MPTP) for PD model induction. The MPTP dose was calculated according to body weight (20 mg/kg/day) and was administered once daily for 14 consecutive days.

After MPTP model induction, model validation was first performed, followed by exploratory omics analyses. Behavioural tests were conducted in 15 Ctrl mice and 15 MPTP-treated mice to assess whether the model had been successfully established. group and one mouse from the MPTP model group were used for TH immunohistochemical validation, and SNpc-enriched midbrain tissue from one mouse in each group was used for single-nucleus RNA sequencing and spatial transcriptomic analysis to explore cell type and pathway alterations in the SNpc region after MPTP treatment. In addition, one mouse from the Ctrl group and one mouse from the MPTP model group was selected for TH immunohistochemical validation. SNpc-enriched midbrain tissue from one mouse in each group was also collected for single-nucleus RNA sequencing and spatial transcriptomic analysis to identify candidate cell type and pathway alterations in the SNpc region after MPTP treatment.

Subsequently, the remaining 28 MPTP-treated mice were randomly assigned to a PD sedentary group (St group, *n* = 14) or a PD exercise group (Ex group, *n* = 14). The remaining Ctrl mice (*n* = 13) were maintained under standard sedentary housing conditions and were not subjected to treadmill exercise. Mice in the Ex group were subjected to 6 weeks of moderate-intensity treadmill exercise, whereas mice in the St and Ctrl groups were kept sedentary under the same housing conditions. After the exercise intervention, behavioural tests were performed in the Ctrl St, and Ex groups. The mice were then euthanized, and tissue samples were collected for subsequent validation experiments, including immunofluorescence staining, biochemical assays, qPCR, Western blotting, transmission electron microscopy, and metabolomic analysis. The sample size used for each experiment is specified in the corresponding Methods Section, Results Section, and figure legends.

The predefined animal exclusion criteria included death during MPTP administration or exercise intervention, failure to show clear PD-like behavioural changes after model induction, severe body weight loss, persistent refusal to run, and obvious injury or illness. No animals met these exclusion criteria during the experiment.

### 2.2. Exercise Intervention

A moderate-intensity aerobic exercise intervention was performed [20]. Mice in the Ex group first underwent a 1-week treadmill adaptation period. During the first 3 days, the treadmill speed was set at 6 m/min and was then increased by 3 m/min per day until a speed of 12 m/min was reached. During the formal intervention, mice exercised at 12 m/min for 60 min/day, 5 days/week, for 6 weeks. The intervention was scheduled between 13:00 and 14:00. During treadmill exercise, mice were continuously monitored for signs of fatigue, refusal to run, abnormal gait, abnormal respiration, obvious stress responses, or reduced exercise capacity. If a mouse was unable to maintain the treadmill speed, remained at the rear of the treadmill for a prolonged period, or showed obvious signs of discomfort, training was paused and the animal was monitored.

### 2.3. Behavioural Analysis

Balance beam test: The Shanghai Xinruan balance beam apparatus was used. The apparatus comprised a starting platform, a target platform, and a central metal beam. During the adaptation phase, mice were placed on the starting platform and were allowed to walk along the balance beam to reach the target platform. This procedure was repeated several times. During the formal test, the time required for the mice to travel from the starting platform to the target platform, walking speed, and number of falls were recorded. Each mouse was tested three times, with a 30 min interval between trials.

Water maze experiment: Each mouse was placed at the starting point of the water maze, and the time required to locate the hidden platform was recorded. Mice were trained for 1 week, with several trials performed each day. After the training period, a spatial probe test was conducted after the platform had been removed. The time spent in the target quadrant where the platform had originally been located and the number of platform area crossings were recorded as measures of spatial memory.

Open-field test: The Shanghai Xinruan open-field testing apparatus was used. The apparatus consisted of an open-topped square enclosure (50 cm × 50 cm × 50 cm), which was divided into central and peripheral areas. The central area was considered an anxiety-related zone that mice tend to avoid. Mice were allowed to acclimatize to the environment for 5 min. During the formal test, mice were placed in the centre of the open field, and their locomotor behaviour was recorded for 300 s.

Elevated plus maze: The elevated plus maze was used to assess anxiety-like behaviour. Each mouse was placed on the central platform facing either an open arm or a closed arm and was allowed to explore freely for 5 min. The number of entries into the open and closed arms and the time spent in each arm were recorded.

### 2.4. Sample Collection

Mice were anesthetized with sodium pentobarbital and then were transcardially perfused with 15 mL of phosphate-buffered saline (PBS). According to the mouse brain atlas, an SNpc-enriched midbrain tissue block was dissected using a brain slicing matrix (slots 13–14; anatomical coordinates of the SNpc: 3.00 mm posterior to bregma, 1.1 mm lateral to the midline, and 4.5 mm below the dura; thickness, approximately 2–3 mm). Deep anesthesia was confirmed by the absence of pedal withdrawal and corneal reflexes.

### 2.5. Conclusion Immunohistochemical Identification of Tyrosine Hydroxylase

Fourteen days after model induction, midbrain tissue was harvested from mice in the Ctrl and PD groups for TH immunohistochemical analysis. Following left ventricular perfusion, the tissue was fixed in 4% paraformaldehyde at 4 °C for at least 24 h. The fixed tissue was embedded in paraffin and sectioned. The sections were heated in a 60 °C oven for 30 min to melt the paraffin and were then deparaffinized and rehydrated using xylene, anhydrous ethanol, and graded ethanol. Endogenous peroxidase activity was blocked with 3% hydrogen peroxide, and the sections were then washed with PBST buffer. Antigen retrieval was performed by boiling the sections in sodium citrate antigen retrieval buffer (pH 6.0) for 10 min. After cooling, the sections were circled with a hydrophobic barrier pen and blocked with 5% BSA. The sections were then incubated with a mouse anti-TH primary antibody (MAB318; Chemicon, Merck KGaA, Darmstadt, Germany; 1:1000). After washing with PBST, DAB substrate was added for colour development, and the reaction was monitored under a microscope. Images of the SNpc region were acquired using an upright optical microscope. Negative control sections were processed in parallel with the primary antibody omitted.

### 2.6. Single-Cell Nuclear Sequencing and Spatial Transcriptomics

For single-nucleus RNA sequencing, midbrain samples were harvested from the 5.3–6.3 mm region according to a mouse brain stereotaxic atlas. A single-nucleus suspension was prepared, and sequencing libraries were constructed using the 10× Genomics platform. Quality-control filtering, dimensionality reduction, clustering, cell annotation, and screening for differentially expressed genes (*p* < 0.05, fold change > 1.5) were then performed. For spatial transcriptomics, midbrain samples were collected using the same anatomical sampling method. Spatial transcriptomic libraries were constructed using the 10× Genomics platform, and Space Ranger software (10x Visium) was used to quantify spots and gene/UMI counts. Data normalization, spatial feature analysis, cell type annotation, and differential gene screening (*p* < 0.05, fold change > 1.5) were performed. The RCTD (Robust Cell Type Decomposition) algorithm was used to decompose cell type composition in the spatial transcriptomic data by using cell type signatures derived from single-nucleus RNA sequencing data.

### 2.7. Immunofluorescence

After fixation, washing, dehydration through graded ethanol, clearing in xylene, and paraffin embedding, the tissue was sectioned into 4 μm sections. The sections were deparaffinized in xylene, rehydrated through graded ethanol, and subjected to antigen retrieval by boiling in sodium citrate buffer. The sections were then washed with PBS, and the tissue area was outlined using a hydrophobic barrier pen. Non-specific staining was blocked with 5% BSA. Primary antibodies used for immunofluorescence included an anti-PLP1 antibody (mouse monoclonal, MA1-90652, 1:100; Invitrogen/Thermo Fisher Scientific, Waltham, MA, USA), an anti-Olig2 antibody (goat polyclonal, AF2418, 8–10 μg/mL; R&D Systems, Minneapolis, MN, USA), and an anti-GPX4 antibody (rabbit monoclonal, ab125066, 1:100–1:400; Abcam, Cambridge, UK). Fluorescent secondary antibodies included donkey anti-mouse IgG Alexa Fluor 488 (A-21202, 1:500–1:1000; Invitrogen/Thermo Fisher Scientific), donkey anti-goat IgG Alexa Fluor 594 (A-11058, 1:500–1:1000; Invitrogen/Thermo Fisher Scientific), and donkey anti-rabbit IgG Alexa Fluor 647 (A-31573, 1:500–1:1000; Invitrogen/Thermo Fisher Scientific). Negative control samples were processed in parallel with the primary antibodies omitted. The sections were then washed again with PBST. The sections were incubated with DAPI solution (1:1000) at room temperature in the dark for 10 min, washed with PBS, and mounted with anti-fade mounting medium. Images were captured using a laser confocal microscope and analyzed using ZEN software (ZEN 3.1). PLP1, Olig2, and GPX4 signals were detected using the corresponding fluorescent secondary antibodies, and nuclei were counterstained with DAPI. For each field of view, single-channel and merged images were acquired for PLP1/Olig2/DAPI or PLP1/GPX4/DAPI staining.

### 2.8. Assay Using a Kit

In accordance with the manufacturer’s instructions for the Iron Assay Kit (BTK079), Malondialdehyde (MDA) Assay Kit, Reduced Glutathione (GSH) Assay Kit (BTK034), and 8-hydroxy-2′-deoxyguanosine (8-OHdG) Assay Kit (ADI-EKS-350), the absorbance values of each sample were measured, and the corresponding concentrations were calculated.

### 2.9. Transmission Electron Microscope

Following sampling, mouse brain tissue was cut into 1 mm^3^ blocks, fixed in 2.5% glutaraldehyde for 24 h, rinsed with PBS, and then post-fixed in 1% osmium tetroxide for 2 h. The tissue was dehydrated through graded ethanol, followed by acetonitrile substitution and infiltration with a mixture of acetonitrile and epoxy resin. The samples were then embedded in pure epoxy resin and polymerized. Ultrathin sections (70 nm) were cut using an ultramicrotome and stained with uranyl acetate and lead citrate. The ultrastructure of OLs was examined using a transmission electron microscope, with particular attention paid to mitochondrial morphology. High-resolution images were captured using electron microscopy software (GMS 3.40).

### 2.10. Non-Targeted Metabolomics

Midbrain tissue was collected after PBS perfusion, rapidly frozen in liquid nitrogen, sonicated, and centrifuged. The supernatant was collected, concentrated, dried, reconstituted in methanol, and filtered before LC-MS analysis. LC-MS analysis was performed using a chromatographic column. Mobile phase A consisted of 0.1% formic acid in water, and mobile phase B consisted of acetonitrile. Gradient elution was applied. Data were processed using XCMS. Candidate metabolites were identified using principal component analysis (PCA) and partial least-squares discriminant analysis (PLS-DA), followed by screening for differentially abundant metabolites and KEGG pathway analysis.

### 2.11. Quantitative Real-Time PCR

Approximately 20 mg of midbrain tissue was lysed for total RNA extraction. After chloroform phase separation, isopropanol precipitation, and ethanol washing, the RNA pellet was dissolved in DEPC-treated water and centrifuged. Reverse transcription was performed according to the manufacturer’s instructions for the cDNA synthesis kit (Table 1). The corresponding primers (Table 2) were added according to the manufacturer’s instructions for the qPCR SuperMix kit. qPCR was performed on a real-time PCR system using a three-step protocol: initial denaturation at 94 °C for 30 s, followed by 40 cycles of 94 °C for 5 s, 55 °C for 15 s, and 72 °C for 10 s. Amplification curves were then obtained. Primer specificity was verified by melting curve analysis, and only products showing a single melting peak were included (Figure S1). Relative gene expression was calculated using the 2^−ΔΔCt^ method, with β-actin used as the internal reference gene.

### 2.12. Western Blot

Approximately 20 mg of substantia nigra tissue from each group was weighed, mixed with 200 µL of RIPA lysis buffer containing protease inhibitors, and homogenized using a high-throughput homogenizer. The homogenate was centrifuged at 12,000 rpm for 15 min at 4 °C, and the supernatant was collected as the protein lysate. Protein concentration was determined using a BCA assay kit, and the loading volume was calculated according to the standard curve. Subsequently, 10% SDS-PAGE gels were prepared, and proteins were separated by electrophoresis. The gel was stained with Quick Blue staining solution to visualize protein yield. Western blotting was then performed. The loading volume was determined based on the gel staining results, followed by electrophoresis, membrane transfer, blocking, primary antibody incubation, secondary antibody incubation, and ECL detection. Protein band intensities were analyzed using ImageJ software (version 1.54u). The primary antibodies used were anti-TH (mouse, BS1600, 1:1000; Bioworld Technology, Nanjing, China), anti-GPX4 (rabbit, BS7323, 1:1000; Bioworld Technology), anti-NRF2 (mouse, BS79743, 1:1000; Bioworld Technology), and anti-β-actin (mouse, MAB48206, 1:1000; Servicebio, Wuhan, China). The corresponding grayscale quantification of Western blot bands is shown in Figure S2.

### 2.13. Statistical Methods

Statistical analyses were performed using SPSS 23.0 (IBM Corp., Armonk, NY, USA), ImageJ, and GraphPad Prism 9 (GraphPad Software, San Diego, CA, USA). ImageJ was used to quantify immunofluorescence images and Western blot bands. All quantitative data are presented as the mean ± SEM. For behavioural data, immunofluorescence quantification, tissue biochemical measurements, qRT-PCR, Western blotting, and relative metabolite abundance, normality was first assessed using the Shapiro–Wilk test, and homogeneity of variance was assessed using Levene’s test. For comparisons among three or more groups, normally distributed data with homogeneous variance were analyzed using one-way ANOVA followed by Tukey’s post hoc multiple-comparison test. Data that did not meet the assumptions of normality or homogeneity of variance were analyzed using the Kruskal–Wallis test followed by Dunn’s multiple-comparison test. For comparisons between two groups, normally distributed data were analyzed using an independent-samples *t*-test, whereas non-normally distributed data were analyzed using the Mann–Whitney U test. For all post hoc pairwise comparisons following omnibus group tests, adjusted *p* values were reported after correction for multiple comparisons. All statistical tests were two-sided, and *p* < 0.05 was considered statistically significant. For untargeted metabolomics, candidate differential metabolites were initially screened using VIP values from the OPLS-DA model in combination with univariate statistical analysis. In the initial analysis, metabolites with VIP > 1 and nominal *p* < 0.05 were considered candidate differential metabolites. To control the risk of false positives caused by multiple comparisons, FDR-adjusted *p* values were further used to assess statistical significance.

## 3. Results

### 3.1. A Mouse Model of Parkinson’s Disease Has Been Successfully Developed

**Figure 1:**
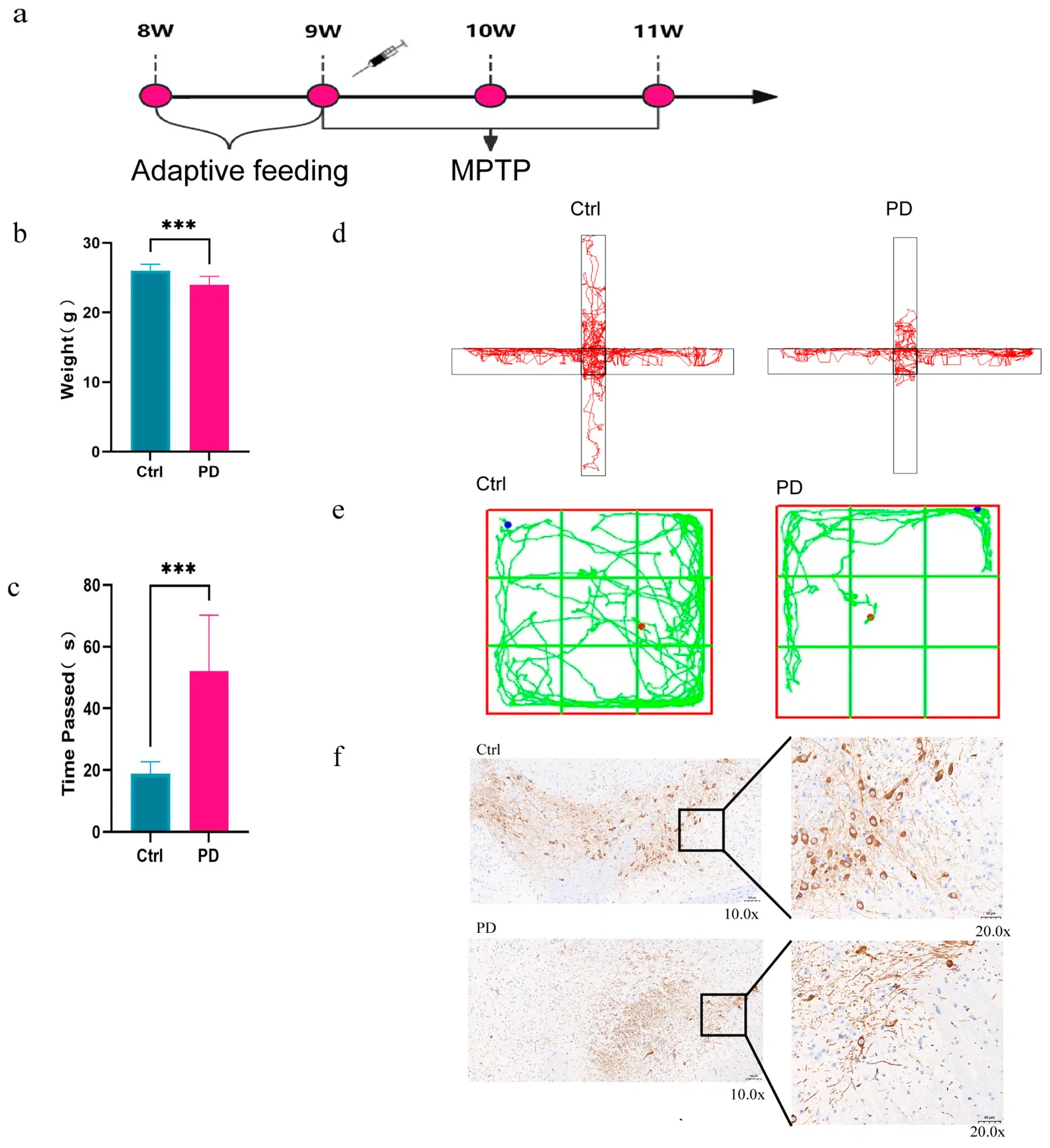
Mouse body weight, behavioural tests and Th immunohistochemistry. Note: (**a**) the timeline for the generation of MPTP-induced mouse models; (**b**) quantitative comparison of body weight; (**c**) dwell time on the balance beam; (**d**) trajectory plots from the open-field test; (**e**): field trials; (**f**) TH staining of the substantia nigra. Data are presented as the mean ± SEM. *n* = 15 animals per group for body weight and behavioural tests. Behavioural tests were repeated three times for each animal, and the average value was used for statistical analysis. Comparisons between groups were performed using an independent-samples *t*-test. TH immunohistochemistry was used for representative model validation (*n* = 1 animal per group) and was not subjected to quantitative statistical analysis. *** indicates *p* < 0.001.

A PD mouse model was established in C57BL/6J mice by intraperitoneal injection of MPTP (Figure 1a). Body weight was significantly lower in PD mice than in control mice (Figure 1b). Behavioural testing revealed that PD mice took significantly longer to traverse the balance beam than control mice (Figure 1c); in the open-field test, the number of wall contacts, distance travelled, and activity trajectories in the open arms of the elevated plus maze were significantly reduced; in the open-field test, the mice showed a preference for the peripheral areas (Figure 1c–e). Th staining of the brain revealed that, compared with normal mice, PD mice exhibited a significant reduction in Th-positive neurons in the SNPC region (Figure 1f).

### 3.2. Single-Cell Sequencing and Spatial Transcriptomics Results

**Figure 2:**
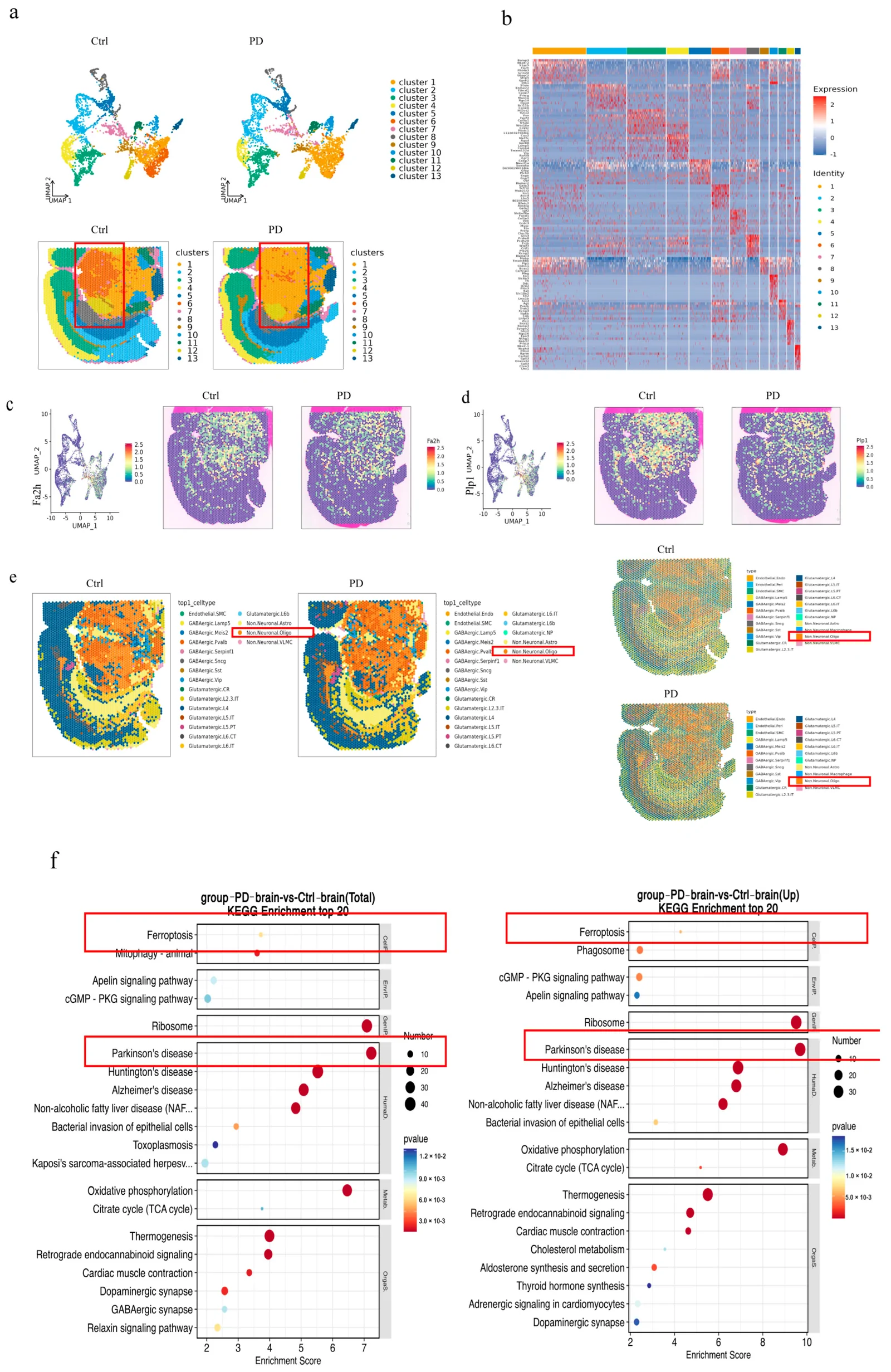
Spatial transcriptomics sequencing and single-cell mapping results. Note: (**a**) a spatial transcriptomic dimension reduction clustering and brain region distribution map; (**b**) a spatial heatmap of gene expression for the top 10 marker genes; (**c**,**d**) spatial visualizations of UMAP clustering results; (**e**) a spatial deconvolution map of RCTD cell types; (**f**) KEGG enrichment results for the control and PD groups in OLs nuclear sequencing. Red box: Changes associated with Parkinson’s disease, oligodendrocytes and oxidative stress. *n* = 1 animal per group was used to screen candidate cell type and pathway alterations (1.2 × 10^−2^).

Single-nucleus RNA sequencing and spatial transcriptomics were used to identify cell type and pathway alterations in the SNpc region after MPTP model induction. Spatial transcriptomics sequencing of wild-type and PD mice revealed that four of the 13 subclusters identified through dimensionality reduction clustering were significantly enriched in the midbrain region (Figure 2a). Differentially expressed genes were screened using the bimodal test, and a heatmap of the spatial expression patterns of the top 10 marker genes (Figure 2b) and a UMAP clustering visualization were generated. These revealed that the classic OL marker genes Nkx6-2, Fa2h, Tmem88b, Plp1 and Cldn11, and compared with normal mice, the expression levels of these genes were significantly reduced in PD mice, exhibiting a trend of spreading towards the periphery, with particularly significant changes observed in Fa2h and Plp1 (Figure 2c,d).

Following single-cell nuclear sequencing, the RCTD algorithm was employed to map the cellular landscape derived from single-cell sequencing onto the spatial transcriptomics results, revealing a similar trend of reduced OL numbers in the midbrain region of PD mice (Figure 2e). Further analysis of intergroup differential expression in OLs, followed by functional enrichment of differentially expressed genes, yielded a KEGG pathway enrichment analysis bubble plot (Top 20) comparing the two groups (Figure 2f). The results indicate that, compared with the Ctrl group, genes associated with ferroptosis and PD-related genes were significantly enriched in OLs from PD mice.

### 3.3. The Effects of 6 Weeks of Treadmill Exercise on Body Weight, Motor Function and Dopaminergic Neurons in Mice with Parkinson’s Disease

**Figure 3:**
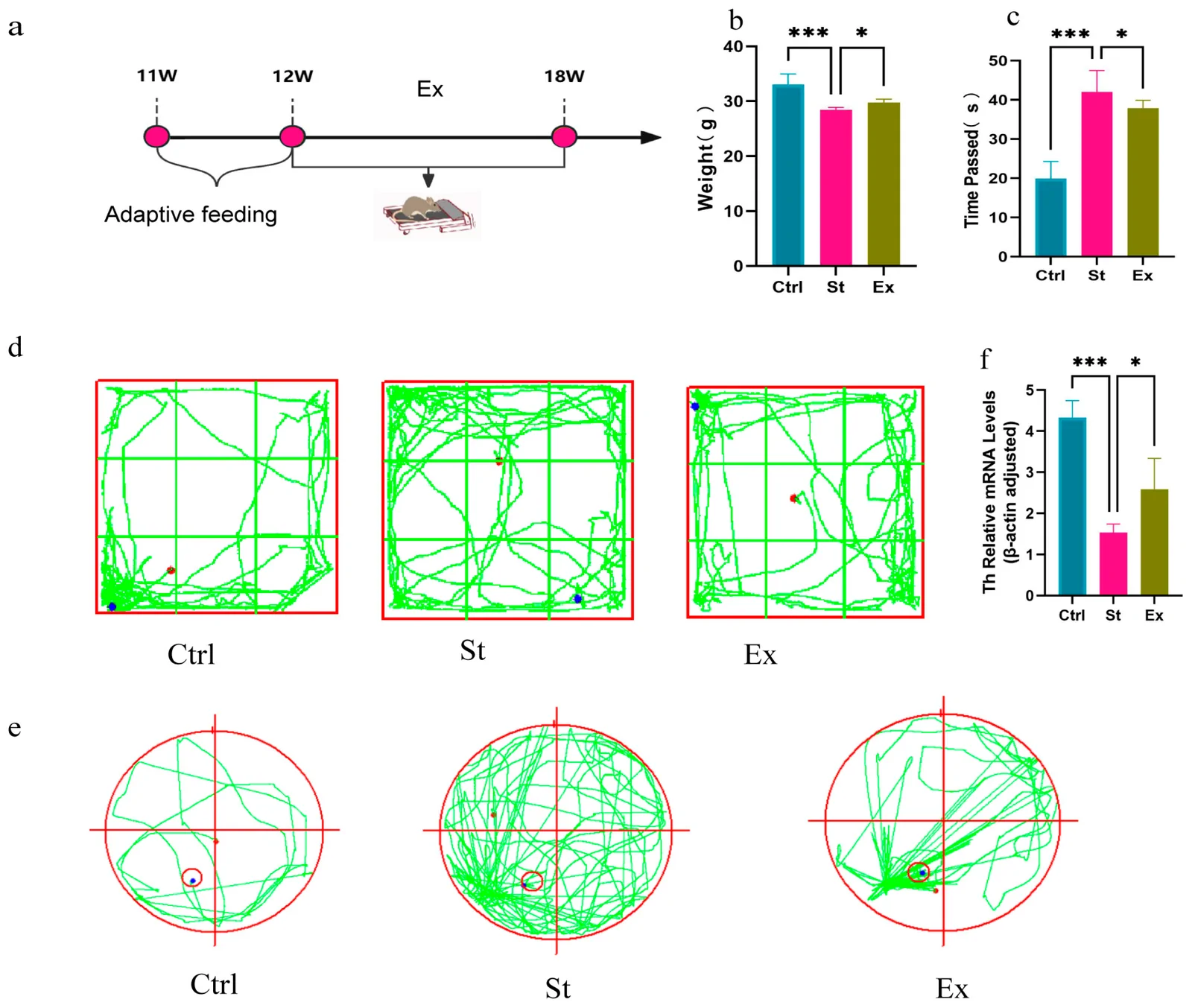
Body weight and behavioural results in mice. Note: (**a**) a graph showing treadmill exercise intervention in 6-week-old mice; (**b**) comparison of mouse body weight; (**c**) comparison of time taken to complete the balance beam test; (**d**) behavioural trajectories in the open-field test; (**e**) water maze test results; (**f**) qRT-PCR and Western blot analysis of Th gene and protein expression levels. Data are presented as the mean ± SEM. *n* = 13 animals per group for behavioural tests; *n* = 6 animals per group for qRT-PCR and Western blot analyses. Each biological replicate represents one animal. Statistical analysis was performed using one-way ANOVA. * indicates *p* < 0.05 and *** indicates *p* < 0.001.

Following six weeks of moderate-intensity aerobic exercise (Figure 3a), body weight and behavioural assessments were conducted on the three groups of mice. The results showed that the body weight of mice in the St group was significantly lower than that of the Ctrl group, whilst the body weight of mice in the Ex group was significantly higher than that of the St group (Figure 3b). The balance beam test revealed that mice in the St group spent significantly longer on the beam than those in the Ctrl group, whilst the Ex group showed significant improvement (Figure 3c). The open-field test demonstrated that the movement trajectories of Ex mice indicated more active exploratory behaviour, with an increase in total distance travelled, whereas mice in the St group tended to circle within the same trajectory (Figure 3d). In the water maze, the number of times mice in the St group passed through the platform quadrant was markedly lower than that of the Ctrl group, but increased following exercise (Figure 3e); qRT-PCR and Western blot results showed that, compared with the Ctrl group, Th gene and protein expression was significantly reduced in the St group, whilst expression levels were significantly increased in the Ex group (Figure 3f).

### 3.4. The Effects of 6 Weeks of Treadmill Exercise on Changes in Oligodendrocytes Within the Substantia Nigra of Mice with Parkinson’s Disease

**Figure 4:**
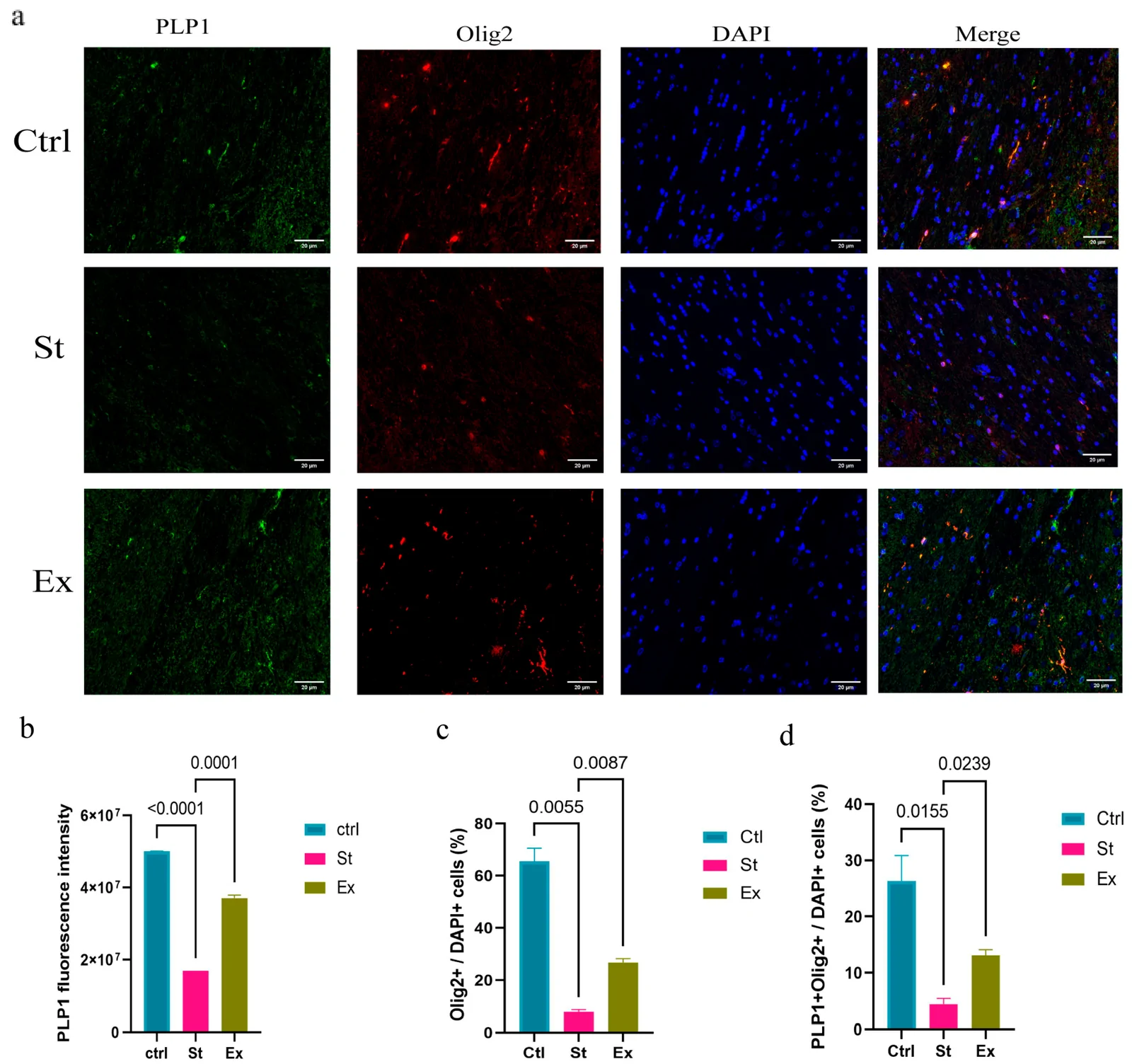
Immunofluorescent expression of PLP1 and Olig2 in the substantia nigra-enriched midbrain tissue of MPTP-induced PD mice. Note: (**a**) Representative immunofluorescence images showing PLP1-positive signals (green), Olig2-positive signals (red), DAPI-stained nuclei (blue), and merged images in the Ctrl, St, and Ex groups; (**b**) quantitative analysis of PLP1 fluorescence intensity; (**c**) quantitative analysis of the proportion of Olig2-positive cells among DAPI-positive cells; (**d**) quantitative analysis of the proportion of PLP1/Olig2 double-positive cells among DAPI-positive cells. scale bar: 20 μm. Data are presented as the mean ± SEM. *n* = 3 animals per group, with the animal used as the statistical unit. Comparisons among the three groups were performed using one-way ANOVA followed by Tukey’s post hoc multiple-comparison test.

Immunofluorescence staining for PLP1 and Olig2 was performed. Compared with the Ctrl group, the St group exhibited reduced PLP1 fluorescence intensity, a lower proportion of Olig2-positive cells, and a decreased proportion of PLP1/Olig2 double-positive cells among total DAPI-positive cells. These findings suggest that MPTP treatment was associated with reduced expression of OL/myelin-related markers in the substantia nigra pars compacta (SNpc). Compared with the St group, the Ex group showed partial recovery of these measures, suggesting that 6 weeks of moderate-intensity treadmill exercise may ameliorate MPTP-induced OL/myelin-related alterations (Figure 4).

### 3.5. Effects of 6 Weeks of Treadmill Exercise on Tissue-Level Ferroptosis-Related Biochemical Markers in SNpc of Mice with PD-like Pathology

**Figure 5:**
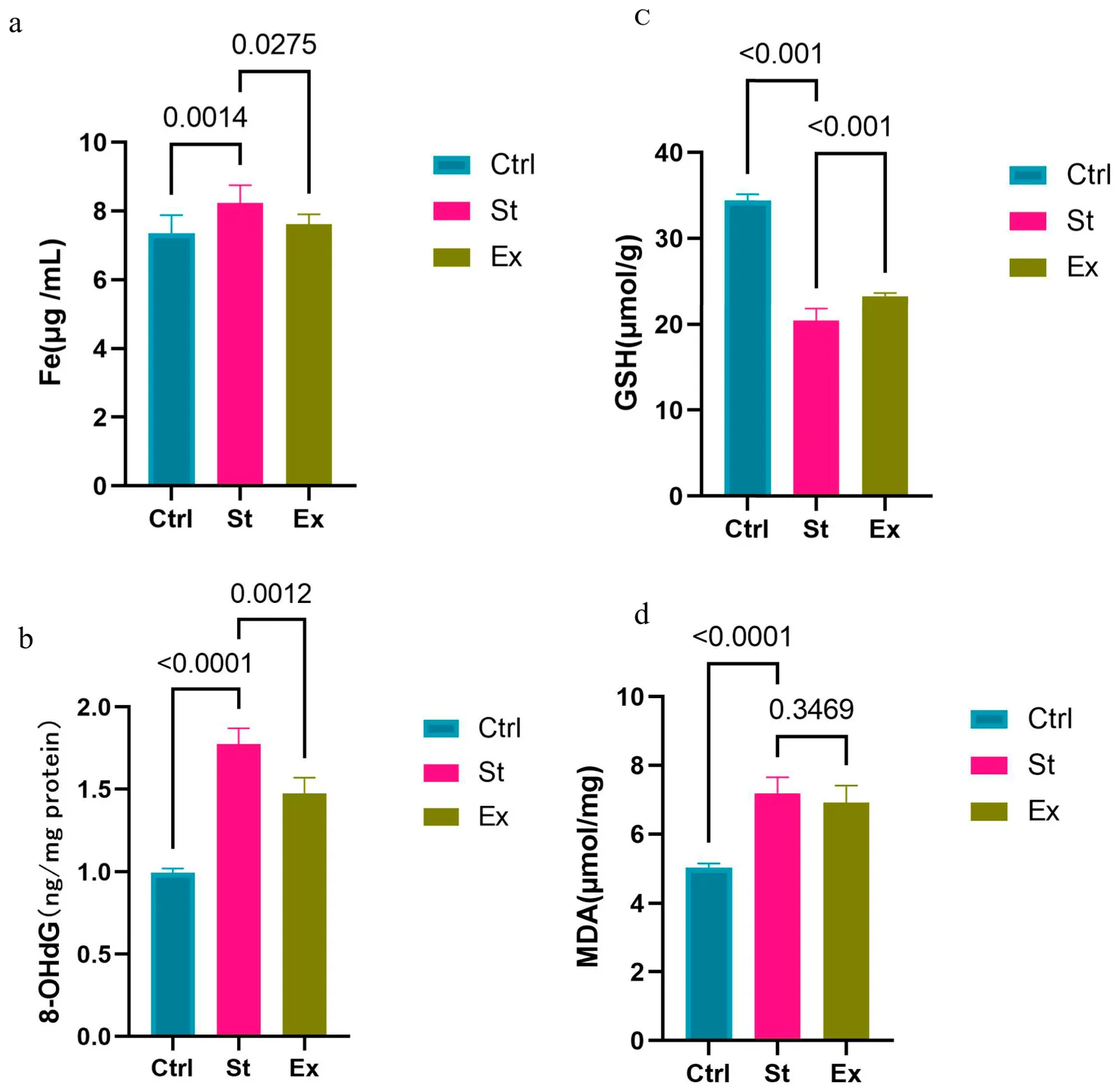
Detection using an iron death-related assay kit. Note: (**a**) the results of the tissue iron content assay; (**b**) the results of the 8-OHdG content assay for the three groups of mice; (**c**) the results of the GSH content assay for the three groups of mice; (**d**) the results of the MDA content assay for the three groups of mice. Data are presented as the mean ± SEM. *n* = 5 animals per group, and each biological replicate represents SNpc-enriched midbrain tissue from one animal. Comparisons among the three groups were performed using one-way ANOVA followed by Tukey’s post hoc multiple-comparison test.

The results of the ferrous iron assay (Figure 5a) showed that, compared with the Ctrl group, the St group exhibited significantly higher levels of ferrous iron in the SNpc tissue of the midbrain; however, following exercise intervention, the Ex group exhibited a significant decrease in ferrous iron levels in the midbrain. Compared with the Ctrl group, the St group exhibited a significant decrease in GSH and MDA levels and a significant increase in 8-OHdG levels in midbrain tissue. Compared with the St group, the Ex group showed a decreasing trend in MDA levels, a significant increase in GSH levels, and a significant decrease in 8-OHdG levels in midbrain tissue.

### 3.6. Effects of 6 Weeks of Treadmill Exercise on Oligodendrocyte Ultrastructure and GPX4-Related Signalling in SNpc of Mice with PD-like Pathology

Changes in ferroptosis within OLs were examined by co-localizing OL marker genes with the ferroptosis marker gene Gpx4.

**Figure 6:**
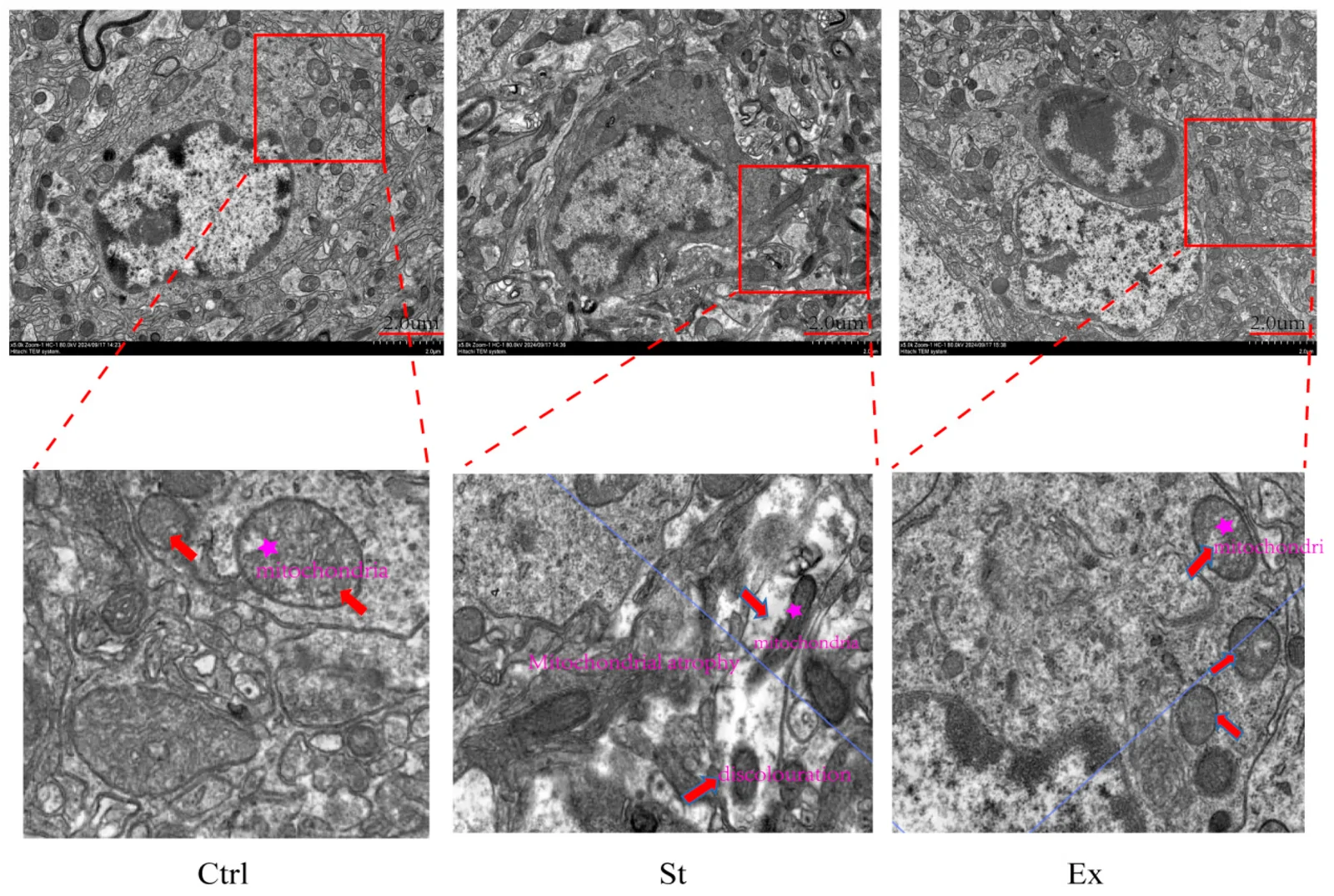
Transmission electron microscopy. Note: Representative transmission electron microscopy images show ultrastructural changes in OLs in the Ctrl, St, and Ex groups. Red boxes indicate enlarged regions, and red arrows indicate representative mitochondrial abnormalities. *n* = 3 animals per group.

Transmission electron microscopy results (Figure 6) showed that, compared with the Ctrl group, OLs in the St group exhibited marked mitochondrial atrophy and vacuolisation, a reduction in mitochondrial cristae, decreased mitochondrial volume, increased membrane density, and blurred or even absent nucleoli, demonstrating distinct pathological morphological features of ferroptosis, whereas the Ex group showed significant alleviation of these pathological morphological manifestations.

**Figure 7:**
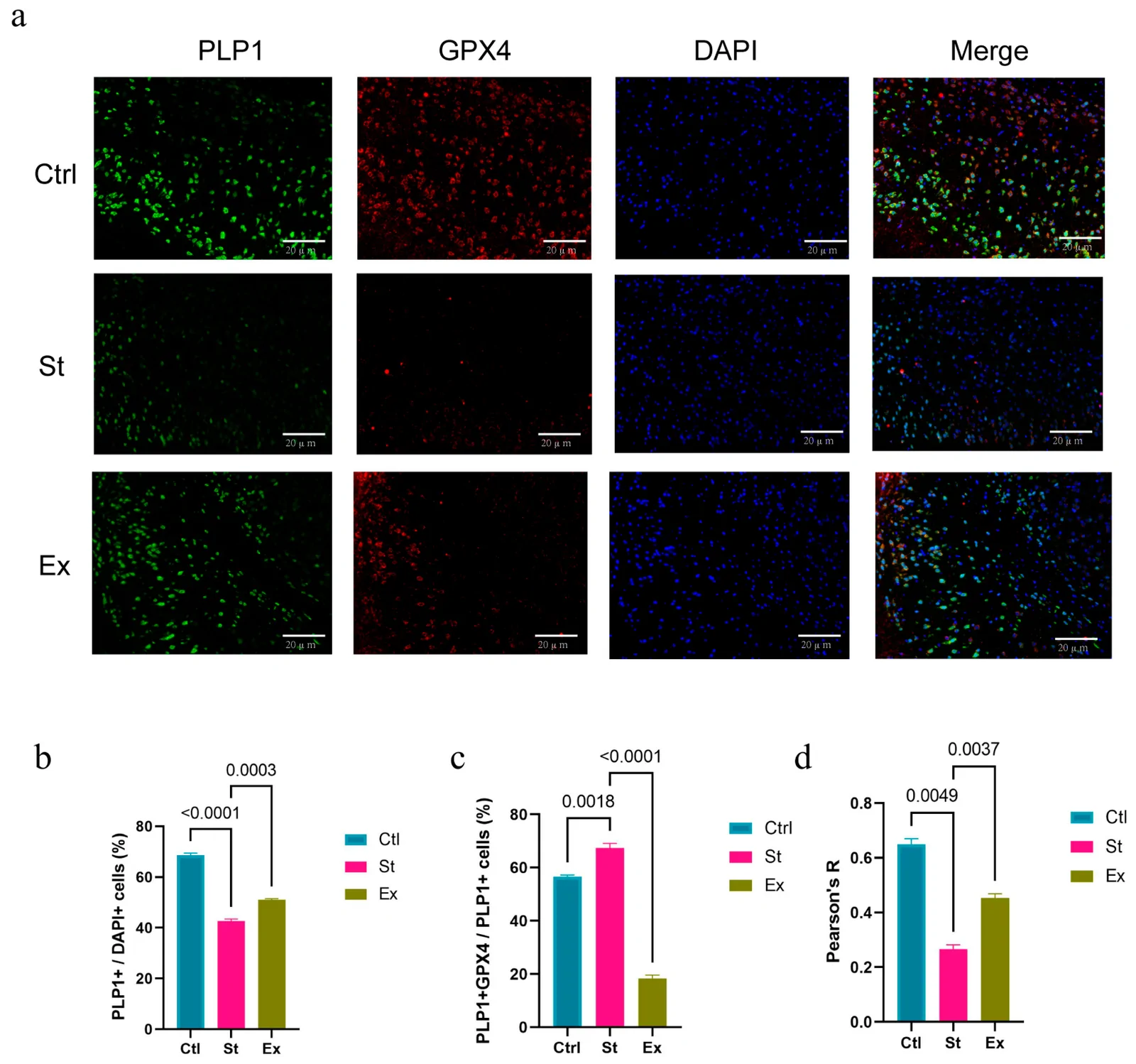
Immunofluorescence co-localization results for Plp1 and Gpx4. Note: (**a**) representative immunofluorescence images showing PLP1-positive signals (green), GPX4-positive signals (red), DAPI-stained nuclei (blue), merged images, and magnified merged views in the Ctrl, St, and Ex groups; (**b**) quantitative analysis of PLP1-positive cell density; (**c**) quantitative analysis of mean GPX4 fluorescence intensity within PLP1-positive areas; (**d**) quantitative analysis of PLP1/GPX4 co-localisation using Pearson’s correlation coefficient. Scale bars: 100 μm for ×20 images and 20 μm for ×40 magnified images. Data are presented as the mean ± SEM. *n* = 3 animals per group, with the animal used as the statistical unit. Comparisons among the three groups were performed using one-way ANOVA followed by Tukey’s post hoc multiple-comparison test.

Immunofluorescence co-localisation results (Figure 7) showed that, compared with the Ctrl group, the St group exhibited a lower density of PLP1-positive cells, reduced mean GPX4 fluorescence intensity within PLP1-positive regions, and a decreased proportion of PLP1/GPX4 double-positive signals. These findings suggest that MPTP treatment was associated with reduced GPX4-related anti-ferroptotic defence in PLP1-positive OLs/myelin-associated regions. Compared with the St group, the Ex group showed partial recovery of these measures, suggesting that treadmill exercise may partially ameliorate the MPTP-associated reduction in GPX4-related anti-ferroptotic signalling within PLP1-positive regions.

### 3.7. Results of Non-Targeted Metabolomics Following 6 Weeks of Treadmill Exercise

**Figure 8:**
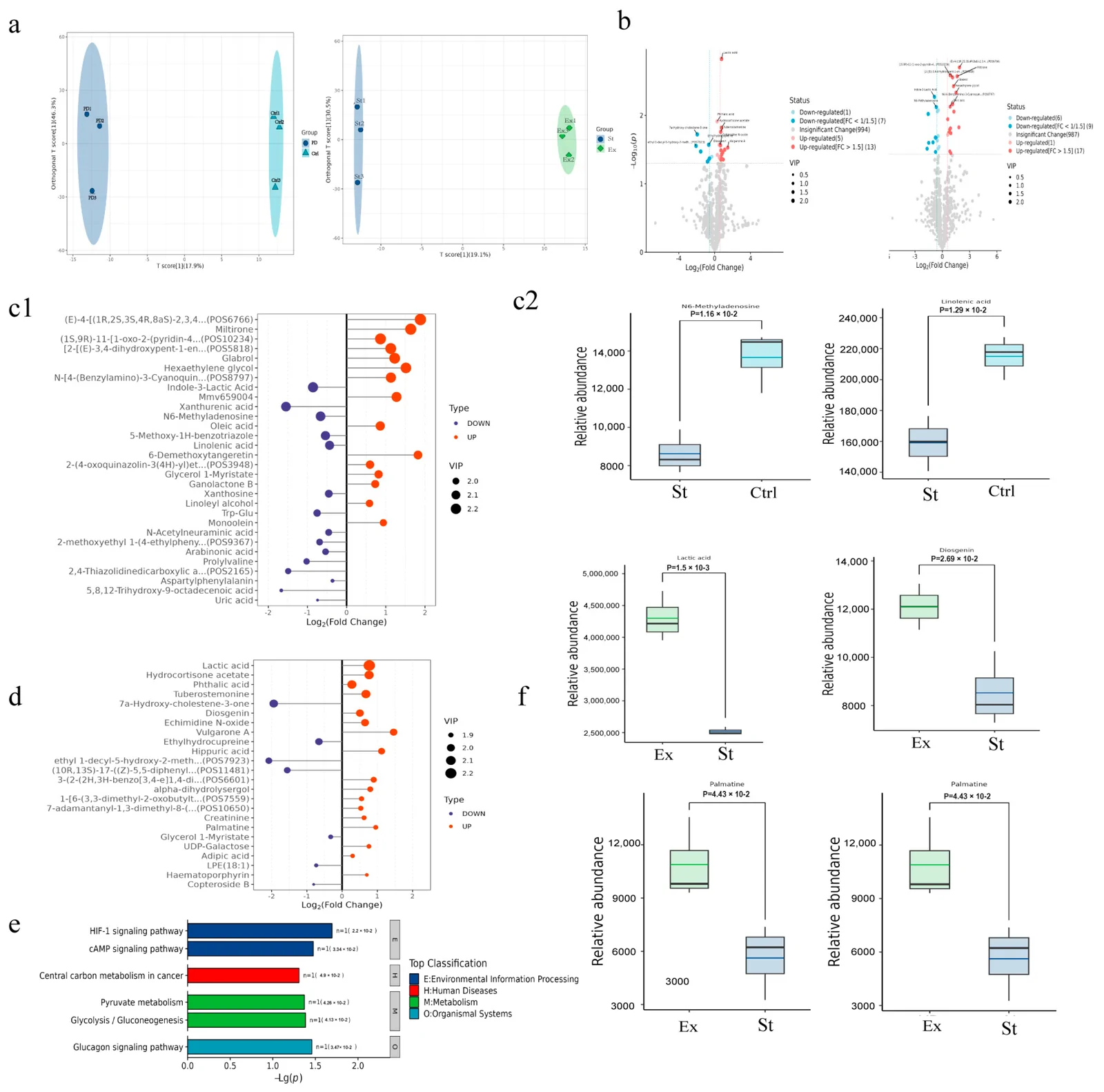
The results of non-targeted metabolomics. Note: (**a**) OPLS-DA plot; (**b**) volcano plot; (**c1**): VIP top 30 fold changes and VIP plot for the comparison group (St. vs. Ctrl). The size of the dots corresponds to the OPLS-DA VIP values; larger dots indicate higher VIP values, with red dots representing upregulation and blue dots representing downregulation. (**c2**) A box plot showing the average expression levels of a specific differentially expressed metabolite across the two groups; (**d**) VIP top 30 fold changes and VIP plots for the comparison group (Ex vs. St); (**e**) a bar chart of significant pathways, showing KEGG pathway enrichment for the comparison group (Ex vs. St); (**f**) a box plot showing the average expression levels of a specific differentially expressed metabolite across the two groups. Non-targeted metabolomic analysis was performed using *n* = 3 biological samples per group, with each sample derived from SNpc-enriched midbrain tissue from one animal. Candidate metabolites were screened based on VIP > 1 and nominal *p* < 0.05, and FDR correction was further applied to assess their robustness.

Orthogonal partial least-squares discriminant analysis (OPLS-DA) showed that the Q^2^ values between groups were greater than 0.5, indicating good predictive performance of the model (Figure 8a). Volcano plots were generated from univariate statistical analysis using the criteria of VIP > 1 and *p* < 0.05 (Figure 8b), with red indicating upregulated metabolites and blue indicating downregulated metabolites. Because untargeted metabolomics involves multiple comparisons, FDR correction was further applied. Some candidate metabolites did not reach the threshold of FDR-adjusted *p* < 0.05. Therefore, these changes were described as candidate differentially abundant metabolites or exploratory metabolic changes to indicate possible metabolic alterations associated with MPTP treatment or exercise intervention. These findings require further validation using targeted metabolomics or independent samples.

St group versus Ctrl group: Based on the exploratory screening criteria, 31 candidate differentially abundant metabolites were identified and displayed in a bubble plot (Figure 8(c1)). Among them, the diterpene quinone compound miltirone, the tryptophan metabolite xanthurenic acid, and uric acid have been reported to be associated with neurodegenerative diseases. In addition, 20 candidate metabolites were related to anti-inflammatory or antioxidant processes and showed a downward trend, including indole-3-lactic acid, xanthurenic acid, 5-methoxy-1H-benzotriazole, xanthosine, and 5,8,12-trihydroxy-9-octadecenoic acid. Because neuroinflammation, oxidative stress, and lipid peroxidation are important features of PD-like pathology and ferroptosis-related stress, these findings suggest that MPTP treatment may be associated with disturbances in metabolic and redox homeostasis. Linolenic acid and N6-methyladenosine (m6A), which may be associated with ferroptosis-related processes, showed a downward trend in the PD group (Figure 8(c2)). Further analysis focused on the potential relationship between these candidate metabolites and OLs. N6-methyladenosine (m6A) and linolenic acid may be associated with OL function and myelin homeostasis. m6A-related regulation has been reported to affect the expression of Mbp and Plp1, thereby influencing myelination and repair. As a polyunsaturated fatty acid (PUFA), linolenic acid is an important precursor of myelin lipids. These results suggest that MPTP treatment may be associated with metabolic disturbances related to OL/myelin function and lipid metabolism. However, these findings remain exploratory observations at the bulk tissue level and cannot directly demonstrate that these changes originate from OLs or directly regulate OL ferroptosis.

Ex group versus St group: Based on the exploratory screening criteria, 24 candidate differentially abundant metabolites were identified. Among them, 11 metabolites were associated with anti-inflammatory or antioxidant functions, and 9 showed an upward trend, including lactic acid, hydrocortisone acetate, and diosgenin (Figure 8d). These changes suggest that exercise intervention may be accompanied by alterations in redox homeostasis and metabolic adaptation. However, they do not directly demonstrate that exercise inhibits ferroptosis through these metabolites. KEGG functional enrichment analysis showed that the enriched pathways included the HIF-1 signalling pathway, cAMP signalling pathway, central carbon metabolism, and pyruvate metabolism (Figure 8e). These pathways are related to energy metabolism, oxidative stress responses, and cellular adaptive regulation. Notably, lactic acid, diosgenin, palmatine, and UDP-galactose may be associated with OL myelination and function. Among them, lactic acid, diosgenin, palmatine, and UDP-galactose showed an upward trend (Figure 8f), suggesting that exercise intervention may be associated with changes in energy metabolism, redox status, and myelin-related metabolic pathways. In addition, phthalic acid showed a downward trend in the untargeted metabolomic analysis, although its biological significance should be interpreted with caution.

### 3.8. Effects of 6 Weeks of Treadmill Exercise on Dopaminergic, Oligodendrocyte-Related, and NRF2/GPX4-Associated Molecular Markers in Mice with PD-like Pathology

**Figure 9:**
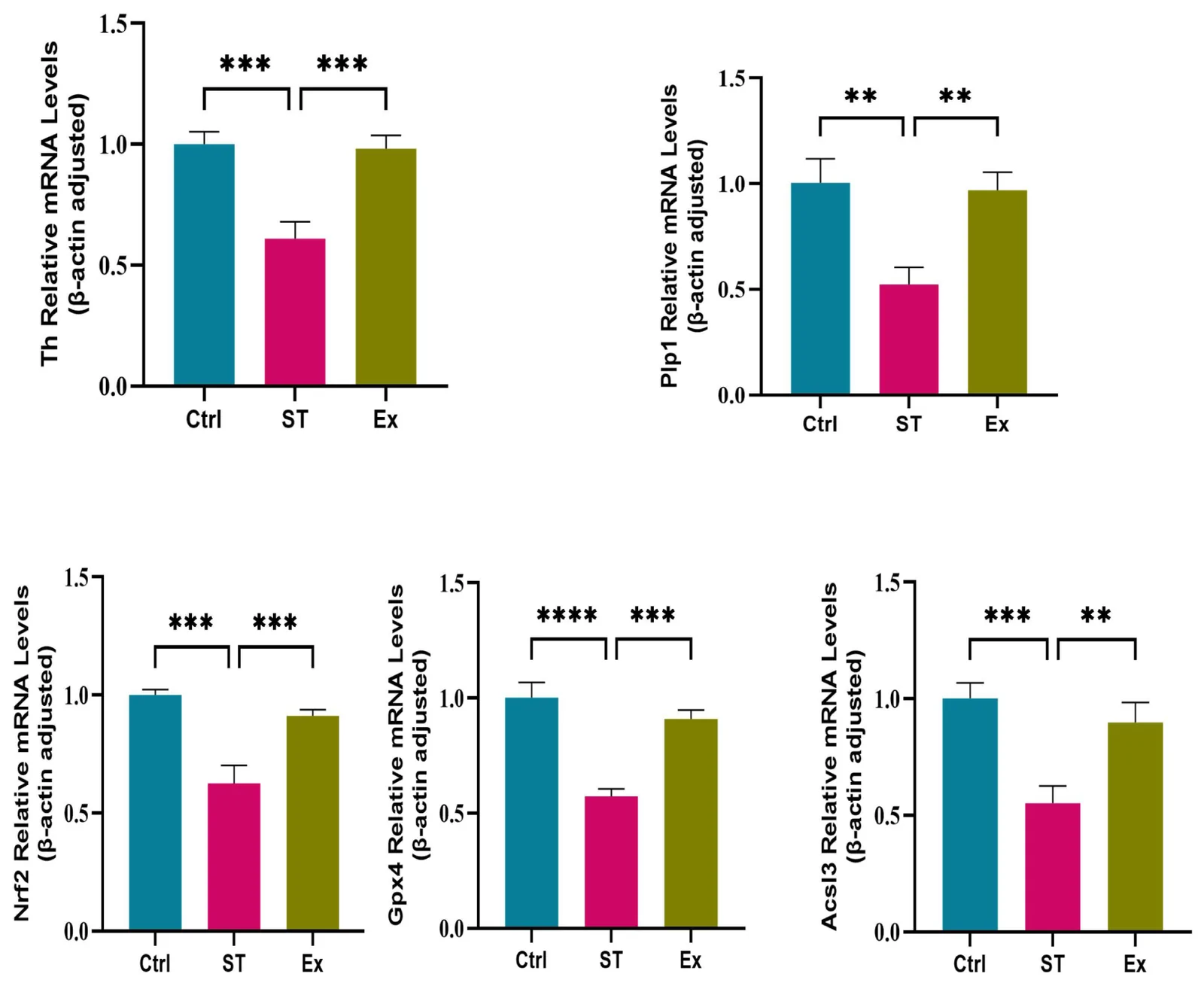
qRT-PCR. Note: Quantitative expression at the transcriptional level of the dopaminergic neuron marker gene Th, the OL marker gene Plp1, and the ferroptosis marker genes Nrf2, Gpx4 and Acsl3d. Data are presented as the mean ± SEM. *n* = 4 animals per group for qRT-PCR analysis, and each biological replicate represents one animal. Comparisons among the three groups were performed using one-way ANOVA followed by Tukey’s post hoc multiple-comparison test. ** indicates *p* < 0.01, *** indicates *p* < 0.001, **** indicates *p* < 0.0001.

PCR results: Compared with the Ctrl group, gene expression levels of Th, a marker enzyme for DA neurons, were significantly reduced in the St group, whereas Th levels increased significantly following a 6-week treadmill exercise intervention. Similarly, in the St group, gene expression levels of Plp1 (a marker gene for oligodendrocytes), and ferrocytosis-related marker genes Acsl3, Nrf2, Gpx4 were significantly reduced; following a 6-week intervention of moderate-intensity aerobic exercise, the DNA levels of Plp1, Nrf2 and Gpx4 in the midbrain of the mice were significantly increased (Figure 9).

**Figure 10:**
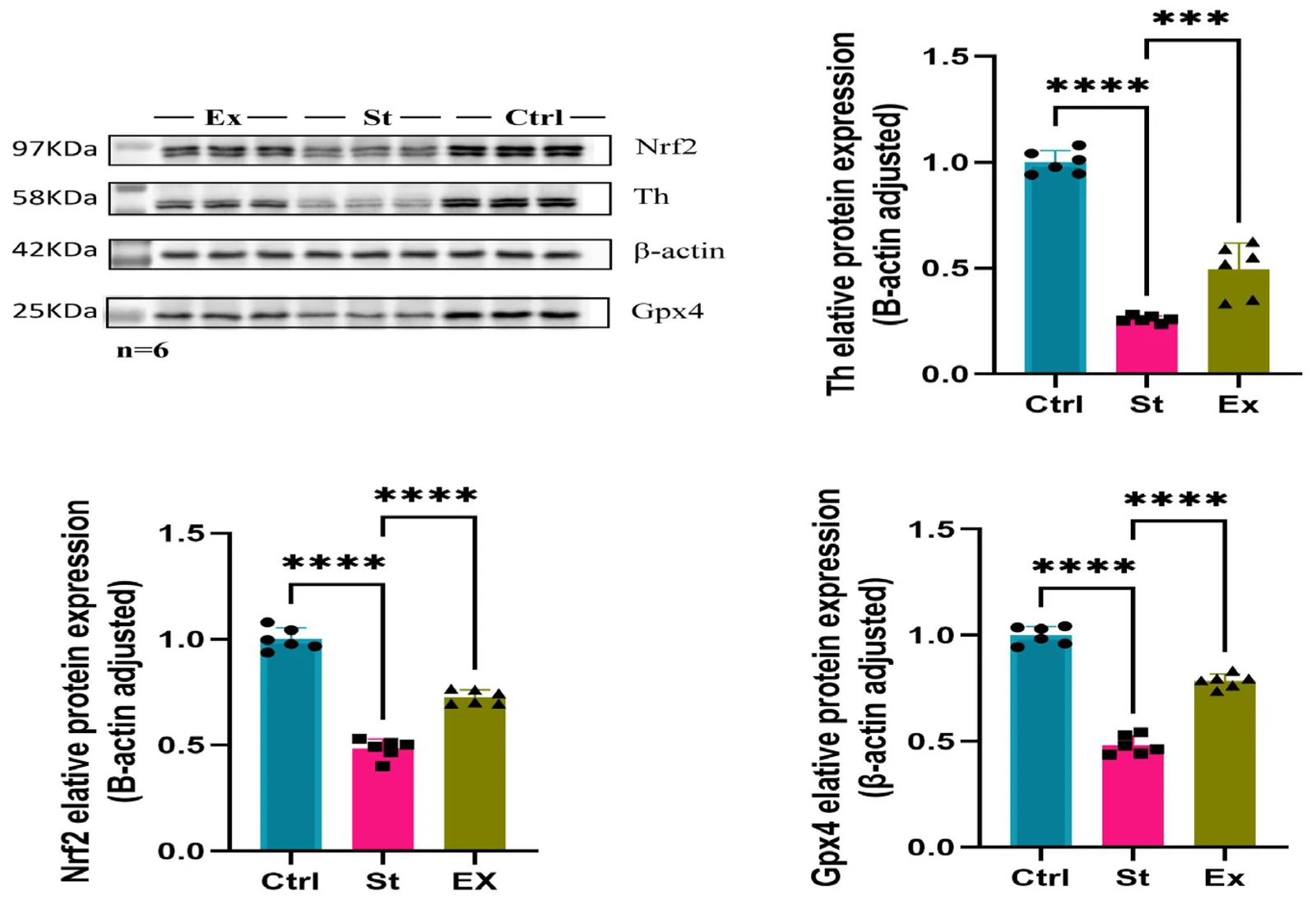
Western blot results. Note: Quantitative expression at the protein level of the dopaminergic neuron marker gene Th and the ferroptosis marker genes Nrf2 and Gpx4; Data are presented as the mean ± SEM. *n* = 6 animals per group for Western blot analysis, and each biological replicate represents one animal. Comparisons among the three groups were performed using one-way ANOVA followed by Tukey’s post hoc multiple-comparison test. *** indicates *p* < 0.001, **** indicates *p* < 0.0001.

Western blot analysis: Compared with the Ctrl group, the protein levels of Th, Nrf2 and Gpx4 in the SNpc of the midbrain of mice in the St group were significantly downregulated (Figure 10).

## 4. Discussion

From the perspective of OLs/myelin injury, our findings suggest that MPTP-induced PD-like pathology was accompanied by reduced OL-associated markers, increased ferroptosis-related stress features, and metabolic abnormalities in the SNpc region, whereas 6 weeks of moderate-intensity treadmill exercise was associated with partial improvement in these pathology-associated alterations.

This study validated the protective effects of aerobic exercise on motor function and DA neurons in PD mice. Treadmill exercise significantly improved motor balance and cognitive function in PD mice, and upregulated Th mRNA and protein expression in the SNpc region, consistent with the findings of Wang et al. [21]. This confirms, at both the behavioural and molecular levels, that exercise can profoundly modulate the intrinsic pathological mechanisms of PD, rather than merely exerting superficial rehabilitative effects.

**Figure 11:**
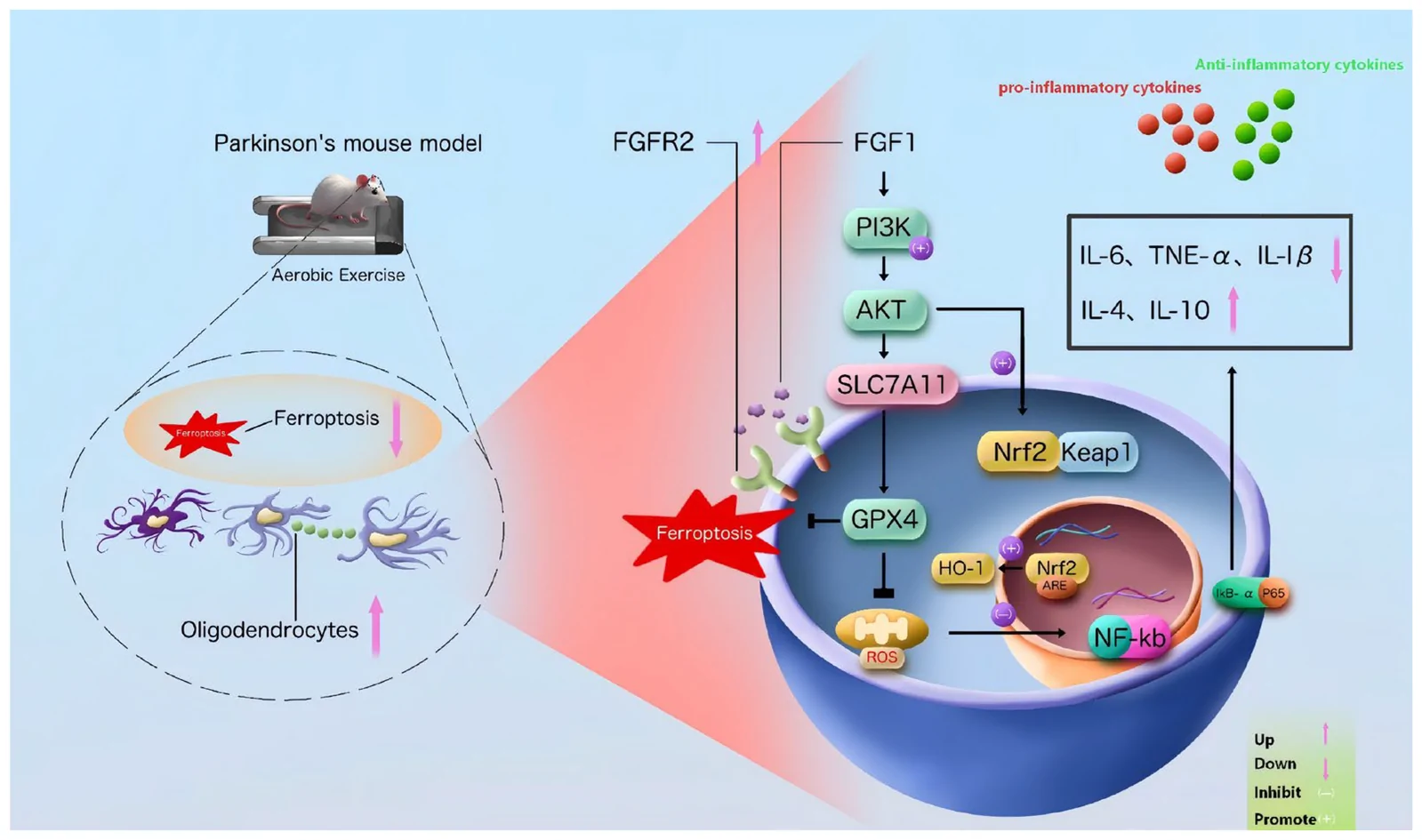
Proposed mechanism by which exercise improves Parkinsonian pathology by alleviating OL injury and ferroptosis-related features.

The effects of exercise on PD-like pathological states may not be limited to OLs and may instead involve coordinated regulation across multiple cell types and pathways. Previous studies have shown that exercise can influence pathological phenotypes in animal models of PD by regulating microglial activation and the NLRP3 inflammasome [21], improving mitochondrial function, enhancing antioxidant defence, modulating brain metabolic status and affecting ferroptosis-related pathways. Recent evidence also suggests that exercise may regulate microglial ferroptosis through the SLC7A11/ALOX12 axis [22,23] and improve neurological function. In addition, exercise may affect reactive astrocyte states by reducing pro-inflammatory and neurotoxic responses while enhancing anti-inflammatory and neuroprotective responses [24]. Therefore, the present study cannot exclude the possibility that microglia, astrocytes, direct protection of DA neurons, improved mitochondrial function, and systemic metabolic adaptations jointly contributed to the exercise-related improvements observed in this model. Within this multicellular pathological context, the present study focused primarily on OL/myelin-associated alterations. Previous studies have reported that changes in OLs may precede pathological alterations in nigral DA neurons [25], suggesting that OLs are not only key effectors of myelination in the central nervous system but also important participants in the pathological cascade of PD [26]. These findings further suggest that early OL abnormalities may not simply be a consequence of DA neuronal degeneration but may also contribute to neuronal injury through bidirectional interactions. In the present study, spatial transcriptomics and cell composition deconvolution analysis showed that the expression of OL-related marker genes was downregulated in the SNpc region of PD mice [7,27], suggesting that MPTP-induced PD-like pathology may be accompanied by OL/myelin-associated abnormalities. This finding is consistent with previous single-cell/single-nucleus transcriptomic studies in patients with PD and MPTP mouse models. For example, Liu et al. performed single-nucleus RNA sequencing of the substantia nigra in MPTP-treated mice and found that the proportion of OLs was significantly reduced after MPTP treatment [28]. Smajić et al. analyzed post-mortem midbrain single-nucleus transcriptomes from six patients with idiopathic PD and control subjects, and also observed a reduction in OL numbers in the PD midbrain, accompanied by upregulation of stress-related genes such as S100B in the remaining OL population [6,29,30]. In addition, previous studies have suggested myelin-related gene dysregulation and altered OL-lineage states in PD-related brain regions. Consistent with these studies, the reduced PLP1- and Olig2-associated signals observed in the present study further support the presence of OL/myelin-associated injury during MPTP-induced PD-like pathology. Moreover, the present study showed that 6 weeks of treadmill exercise increased OL numbers in the SNpc region of PD mice, which is consistent with previous findings that exercise promotes OLs precursor cell (OPC) differentiation and accelerates myelin repair [31,32,33,34]. These results suggest that exercise intervention may improve MPTP-induced OL/myelin-associated abnormalities. However, these findings should still be interpreted as evidence of OL/myelin-associated alterations and cannot, by themselves, prove that OL ferroptosis is the primary driver of PD pathology or exercise-associated protection (Figure 11).

OLs may influence the status of DA neurons by maintaining myelin integrity, supporting axonal metabolism, and regulating the local inflammatory and oxidative stress microenvironment. Previous studies have shown that OL-lineage cells are in spatial contact with the somata and axons of nigral DA neurons [35] and that OL–axon metabolic coupling plays an important role in axonal health. After OL injury, myelin integrity and metabolic support may be reduced, accompanied by disrupted iron homeostasis, increased lipid peroxidation, and decreased GPX4/NRF2-mediated antioxidant defence [36], thereby increasing the susceptibility of DA neurons to oxidative stress injury. Recent studies also suggest that OLs may participate in PD-related neurodegenerative changes through inflammatory pathways such as the PSAP-GPR37-IL-6 axis [31]. Therefore, the decreased OL markers and enhanced ferroptosis-related stress observed in this study may indicate a glial microenvironment unfavourable for DA neuron maintenance; however, OL-DA neuron interactions still require further experimental validation.

Ferroptosis in OLs is a novel mechanism underlying PD pathogenesis and a key target for exercise intervention, as revealed in this study. Single-cell sequencing of OLs demonstrated that ferroptotic pathways are significantly enriched in OLs from PD mouse models. As myelin-producing cells, OLs require iron for myelin formation and the activity of metabolic enzymes [37]. They are the cell type with the highest iron content in the central nervous system. The myelin synthesis process readily generates ROS, and the low GSH content in OLs themselves further exacerbates this effect [11], rendering them highly susceptible to ferroptosis. A potential mechanism suggests that following demyelination, OLs may release large amounts of their own iron into the neuronal iron pool, thereby exacerbating neuronal ferroptosis. This implies that OLs may influence communication with DA neurons through their own reactions, acting as an early trigger for DA neuron inactivation rather than merely a downstream response to stress. Furthermore, ferroptosis and neuroinflammation can form a vicious cycle [38,39], synergistically mediating the progression of PD pathology, thereby corroborating the pivotal role of OL ferroptosis in PD. Future research may further elucidate the mechanisms of iron metabolism interaction between OLs and DA neurons, and explore specific regulatory approaches targeting OL ferroptosis.

The present findings provide further insight into potential mechanisms through which exercise may modulate ferroptosis-related processes in OLs. By integrating transcriptomic, morphological, molecular, and metabolomic evidence, the results suggest that moderate-intensity aerobic exercise was associated with reduced OL/myelin-related injury and ferroptosis-related stress in mice with PD-like pathology, together with improvements in DA neuronal markers and neuropathological phenotypes. Exercise intervention was also accompanied by alterations in NRF2/GPX4-associated antioxidant signalling and several oxidative stress-related measures, suggesting that this pathway may participate in exercise-related antioxidant adaptation. Furthermore, exploratory untargeted metabolomics identified exercise-associated changes in candidate metabolites and pathways related to inflammatory and antioxidant processes, energy metabolism, and myelin-associated functions. These findings provide exploratory evidence of tissue-level metabolic adaptations after exercise intervention. Integrating the metabolomic and transcriptomic findings, our results suggest a potential association among OL injury, ferroptosis-related lipid metabolic disturbances, and exercise-responsive metabolic changes. Reduced GPX4/NRF2-associated antioxidant signalling may indicate a decreased capacity to clear lipid peroxides, whereas changes in PUFA/linolenic acid-related metabolites may influence cellular susceptibility to lipid peroxidation. After exercise intervention, alterations in lactate and pyruvate metabolism, HIF-1 signalling, and central carbon metabolism may reflect improved energetic support within the glial-neuronal unit. In addition, UDP-galactose and several lipid-related metabolites are associated with myelin membrane synthesis and OL function. Together, these findings suggest a possible convergence among OL/myelin injury, ferroptosis-related lipid metabolic dysregulation and metabolic stress in MPTP-induced PD-like pathology, and indicate that exercise intervention may partially ameliorate this abnormal metabolic state. Previous metabolomic studies have also reported changes in glutamate-glutamine metabolism, N-acetylaspartate, myo-inositol and taurine in the striatum and substantia nigra after MPTP exposure, suggesting disturbances in neurotransmitter cycling, glial responses, oxidative stress and osmotic regulation. It should be noted that metabolomic analysis in the present study was performed using bulk SNpc-enriched midbrain tissue. Therefore, these findings should be interpreted as tissue-level supportive and exploratory evidence, rather than direct evidence that these metabolic changes originate from OLs or independently prove OL-autonomous ferroptosis. Future studies combining OL-specific isolation, targeted metabolomic assays and functional intervention experiments are required to further validate this possibility.

In conclusion, this study extends the conventional focus of PD research by providing complementary evidence from the perspective of OL/myelin-related alterations. The findings contribute to an improved understanding of MPTP-induced PD-like pathology and the effects of exercise intervention, suggesting that OL/myelin injury and ferroptosis-related stress may be involved in PD-like pathological processes and may represent one of several components influenced by exercise. These results offer an additional perspective on the mechanisms underlying PD pathology.

This study still has several limitations that should be addressed in future work. First, the biochemical assays in this study were mainly performed using bulk SNpc-enriched midbrain tissue; therefore, the results reflect ferroptosis-related changes at the tissue level. Together with PLP1/GPX4 immunofluorescence co-localisation and ultrastructural observations of OLs, these findings suggest that ferroptosis-related stress may be involved in MPTP-induced OL/myelin injury in the substantia nigra. However, they do not demonstrate that this process is OL-autonomous. Future studies using cell sorting, OL-specific tracing and functional intervention experiments are needed to further validate this possibility. Second, A healthy exercise control group was not included in this study. Because exercise itself can influence metabolism, oxidative stress, inflammatory responses and neuroimmune status even in healthy animals, the current experimental design cannot fully distinguish exercise-related improvements under PD-like pathological conditions from general exercise-induced adaptations. Nevertheless, compared with the MPTP sedentary group, the MPTP exercise group showed improvements in OL/myelin-associated markers and ferroptosis-related stress, suggesting that exercise intervention may exert regulatory effects within the PD-like pathological microenvironment. Future studies should include a healthy exercise control group to further clarify the extent to which these changes are specific to the PD pathological state. Some histological and ultrastructural validation experiments were performed with relatively small sample sizes. Future studies should repeat these analyses using larger sample sizes and independent animal cohorts to improve statistical power and the robustness of the findings.

## 5. Conclusions

In the SNpc-enriched midbrain region of MPTP-induced PD mice, OL-associated markers were reduced and accompanied by enhanced ferroptosis-related pathways, oxidative stress and inflammatory responses. Six weeks of moderate-intensity treadmill exercise partially restored OL-associated marker expression, improved neuropathological phenotypes, and attenuated ferroptosis-related oxidative stress and inflammatory features. These findings suggest that OL injury and ferroptosis-related stress may be involved in PD-related pathological changes, and that exercise intervention may partially ameliorate these abnormalities within this pathological context.

## Figures and Tables

**Table 1:** cDNA synthesis protocol.

Component	Volume
Total RNA/mRNA	≤1 μg/≤100 ng
5×Trans Script^®^ Uni All-in-One SuperMix for qPCR	4 µL
gDNA Remover	1 µL
RNase-free Water	Variable
RNase-free Water	20 µL

**Table 2:** Target gene and primer sequences.

	Forward	Reverse
Th	CCATTGGAGGCTGTGGTATT	GGGTGGTACCCTATGCATTTAG
Nrf2	GCCTTACTCTCCCAGTGAATAC	CTCCCAAATGGTGCCTAAGA
Gpx4	CACATGGTCTGCCTGGATAA	GGAAGGCCAGGATTCGTAAA
Acsl3	GGCTGTGCACTGGAGATATT	GTCTGCCTGGTAGTGTGTTT
β-Actin	GAGGTATCCTGACCCTGAAGTA	GCTCGAAGTCTAGAGCAACATAG

## Data Availability

The data used to support the findings of this study are available from the corresponding author upon request.

## Supplementary Materials

The following supporting information can be downloaded at: https://www.mdpi.com/article/10.3390/brainsci16090904/s1.

## Abbreviations

The following abbreviations are used in this manuscript:

MPTP	1-Methyl-4-phenyl-1,2,3,6-tetrahydropyridine hydrochloride
PD	Parkinson’s disease
SNpc	Substantia nigra pars compacta
COMT	Catechol-O-Methyltransferase
NRF2	Nuclear factor (erythroid-derived2)-like 2
GPX4	Glutathione peroxidase 4
FSPI	Ferroptosis suppressor protein 1
PUFA	Polyunsaturated fatty acid
PLOOH	Phospholipid hydroperoxide
OLs	Oligodendrocytes
OPCs	Oligodendrocyte Precursor Cells
DA	Dopamine
a-Syn	a-Synuclein
iPSC	Induced pluripotent stem cell
OXPHOS	Oxidative phosphorylation
6-OHDA	6-hydroxydopamine
MDA	Malondialdehyde
SOD	Superoxide Dismutase
GSH	Glutathione
ROS	Reactive oxygen species
